# Alternate Directed Anthropogenic Shifts in Genotype Result in Different Ecological Outcomes in Coho Salmon *Oncorhynchus kisutch* Fry

**DOI:** 10.1371/journal.pone.0148687

**Published:** 2016-02-05

**Authors:** Rosalind A. Leggatt, L. Fredrik Sundström, Wendy E. Vandersteen, Robert H. Devlin

**Affiliations:** Centre for Aquaculture and Environmental Research, Fisheries and Oceans Canada, 4160 Marine Drive, West Vancouver, BC, V7V 1N6, Canada; Ghent University, BELGIUM

## Abstract

Domesticated and growth hormone (GH) transgenic salmon provide an interesting model to compare effects of selected versus engineered phenotypic change on relative fitness in an ecological context. Phenotype in domestication is altered via polygenic selection of traits over multiple generations, whereas in transgenesis is altered by a single locus in one generation. These established and emerging technologies both result in elevated growth rates in culture, and are associated with similar secondary effects such as increased foraging, decreased predator avoidance, and similar endocrine and gene expression profiles. As such, there is concern regarding ecological consequences should fish that have been genetically altered escape to natural ecosystems. To determine if the type of genetic change influences fitness components associated with ecological success outside of the culture environments they were produced for, we examined growth and survival of domesticated, transgenic, and wild-type coho salmon fry under different environmental conditions. In simple conditions (i.e. culture) with unlimited food, transgenic fish had the greatest growth, while in naturalized stream tanks (limited natural food, with or without predators) domesticated fish had greatest growth and survival of the three fish groups. As such, the largest growth in culture conditions may not translate to the greatest ecological effects in natural conditions, and shifts in phenotype over multiple rather than one loci may result in greater success in a wider range of conditions. These differences may arise from very different historical opportunities of transgenic and domesticated strains to select for multiple growth pathways or counter-select against negative secondary changes arising from elevated capacity for growth, with domesticated fish potentially obtaining or retaining adaptive responses to multiple environmental conditions not yet acquired in recently generated transgenic strains.

## Introduction

Artificial selection has been used throughout much of human history to enhance desirable traits (e.g. growth rate) in animals and plants. During the domestication process, the genetic shifts imposed on a strain to obtain the desired phenotype are generally not known, and can be accompanied by other unintentional secondary phenotypic and/or genetic changes. More recent developments in biotechnology (i.e. transgenesis) can often result in similar phenotypic changes as domestication, but on a much shorter time frame [1]. For example, selection for fast growth rate in various fish species increases growth, on average, 12.5% per generation (see [2]), with 7% per generation in coho salmon (*Oncorhynchus kisutch*, Walbaum, [3]), whereas insertion of a growth hormone (GH) transgene can result in a many-fold increase in growth rate in only 1–2 generations (see [4]).

The divergent phenotypes of domesticated and GH transgenic fish from wild type have generated significant concern regarding effects they may have should they enter natural ecosystems [4]. Escape of domesticated salmon from aquaculture facilities have recorded historic and ongoing impacts to wild populations through competition and hybridization (e.g. [5–8]). Potential use of transgenic fish for commercial production represents an emerging risk to natural fish populations and ecosystems, but due to current strict regulations regarding transgenic fish biocontainment, no fully natural studies have been undertaken on potential hazards (i.e. potential to cause effects to natural ecosystem components) of these fish. Ecological hazards of transgenic fish in natural ecosystems could potentially be estimated using a comparator with similar growth capacity–specifically domesticated salmon. However, whether parallel ecological consequences may arise from domestication and GH transgenesis in fish has not yet been directly compared.

Two fitness factors that can influence persistence in natural ecosystems, and hence allow for interactions with components of those ecosystems, are growth and survival. Domesticated and GH transgenic fish are both well established to have inheritable enhanced growth rates in culture conditions. Enhanced growth rates have also been reported in many studies in natural (domesticated only, [8–11]) and semi-natural environments [12–19], relative to wild-type fish, although to a lesser degree than observed in culture. However, in some studies, transgenic and domesticated fish can have equal or lesser growth than wild fish, particularly when food abundance is very low and/or predators are present at time of emergence (transgenic fish, [16, 17, 20, 21]), and in some seasons or life stages seasons (domesticated fish, [8, 11], see [22]).

In addition to elevated growth, domestication and GH transgenesis can result in secondary behavioural changes that can influence growth, survival, and ecological effects in non-culture conditions. Both groups of fish are reported in some circumstances to be more aggressive and bold, have more active foraging behaviour, and reduced predator avoidance behaviour relative to wild-type fish [9, 12, 13, 15, 23–30]. This can result in growth-enhanced fish being dominant over wild-type fish in paired contests [9, 31–33], although in some studies wild-type fish were dominant [12, 33] or had equal dominance [28] over domesticated fish, particularly if wild-type fish had residency advantage. As well, GH transgenic Atlantic salmon (*Salmo salar* L.) first-feeding fry did not dominate wild fry, possible due to lack of GH transgene activation at this stage in this transgenic model [34, 35]. Despite increase in dominance, survival of domesticated fish is often, but not consistently, lower than that of wild-type fish in naturalized conditions (e.g. [19], see [22]). This is hypothesized to be due to lower predator avoidance behaviour, although domesticated Atlantic salmon juveniles did not differ from wild type in susceptibility to an artificial predator [36], and have been reported to have higher survival than wild-type fish in conditions of size-selective predation [14]. The reported effects of GH transgenesis in terms of survival in semi-natural conditions are also mixed, where transgenic salmonid fry had enhanced survival in one study [16], lower survival when stressful conditions (i.e. limited food or presence of salmonid predators) were present at time of emergence [20, 21], and equal survival if stressful conditions were introduced later or were not present [16, 17, 20, 21, 37]. As well, dominance in laboratory conditions in salmonids does not necessarily translate to dominance or increased fitness in natural or unpredictable conditions [38–40], further complicating the determination of ecological consequences of accelerated growth and aggression.

While the overall patterns of growth and behaviour are similar between GH transgenic and domesticated fish, it is not fully understood whether the mechanisms that control these phenotypes are similar between the two groups of fish. The scale of engineered genetic change through transgenesis is generally small (i.e. insertion of a single gene with modified expression) and secondary phenotypic effects arise primarily from elevated GH expression. In contrast, the genetic changes brought about by artificial selection involve more genetic loci, and secondary changes may be due to compensatory or unintentional selection for other traits, as well as secondary effects of the desired phenotypic change. Despite these different mechanisms for accelerated growth, gene expression responses of transgenic and domesticated strains are highly correlated in laboratory conditions [41], [42], although whether this is a reflection of similar mechanisms controlling growth, or of similar consequences of accelerated growth, has not been determined. To determine if type of genetic change influences whole-animal phenotypic effects important for ecological success, we examined whether accelerated growth under culture conditions produced by two different genetic processes, GH transgenesis and domestication, may be similarly transferable to complex nature-like environments under competition and predation. For this we examined growth and survival of GH transgenic, domesticated, and wild-type coho salmon fry reared in semi-natural stream tanks with or without predation, as well as in standard culture conditions.

## Materials and Methods

**Fig 1:**
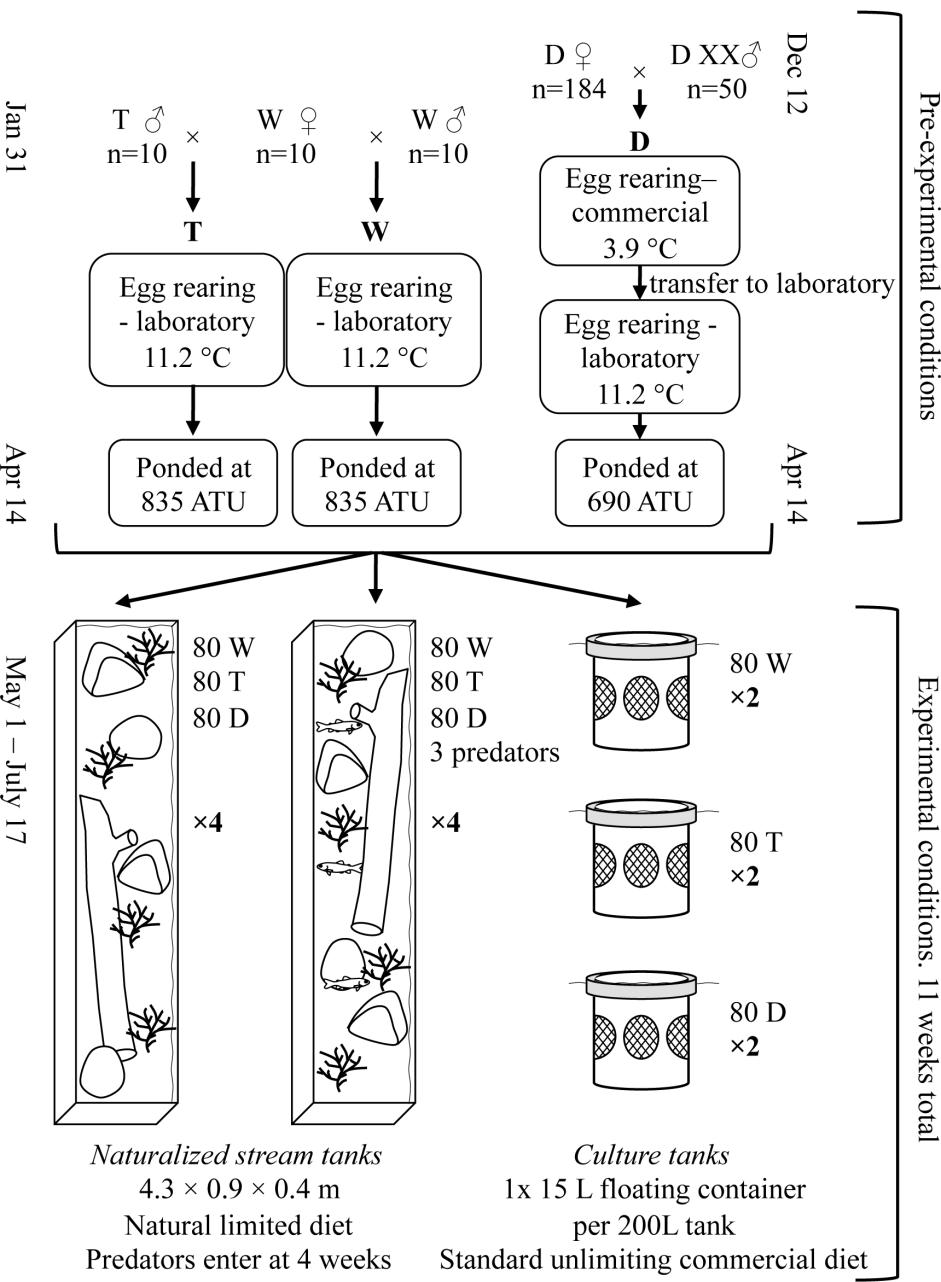
Experimental Design. Pre-experimental conditions (crosses and culture conditions), and experimental design comparing wild-type (W), growth hormone transgenic (T), and commercially obtained domesticated (D) coho salmon (all-female D fish were produced from genotypically female (XX) males) reared in culture or naturalized stream tanks with or without rainbow trout smolt predators. Rainbow trout smolt predators were introduced at 4 weeks, and prior to this fish in predator tanks were exposed to a model heron predator attack at random times per day, 5 days per week. ATU = accumulated thermal units.

Experiments took place at Fisheries and Oceans Canada (DFO) Centre for Aquaculture and Environmental Research (CAER) laboratory, West Vancouver, BC. Approval and permit for this experiment was granted by the Pacific Region Animal Care Committee (Permit number: 14–011). Growth hormone transgenic (T) coho salmon (*Oncorhynchus kisutch* Walbaum) were created by microinjection of a OnMTGH1 transgene construct (GH1 transgene coupled to a metallothionein-B promoter, both from sockeye salmon) into newly fertilized eggs [43], and fish used were produced from a single founding individual (M77 strain, [44]). The strain was created in and backcrossed at each generation to wild-reared coho salmon obtained from the DFO Chehalis River Hatchery, Agassiz, BC. Transgenic fish were produced by single-pair crosses of ten males homozygous for the OnMTGH1 gene to ten wild-type Chehalis River females (see Fig 1 for cross structure and pre-experimental conditions). Wild-type (W) coho salmon were produced from ten Chehalis River hatchery males single-pair crossed to the same females used to produce T fish. The Chehalis River strain is a mixed hatchery/wild population with hatchery production since 1984, and it is expected that ancestors of fish used were a mix of those with juvenile rearing in hatchery and in natural conditions. T and W fish were fertilized on Jan 31, 2014, and maintained in Heath stacks fed with well water (11.2 ± 0.0°C) until first feed. Domesticated coho salmon (D) were an all-female group obtained from a stock population (184 females batch-crossed to 50 males) sourced from a local commercial aquaculture facility (Target Marine Hatcheries, Sechelt, BC). Eggs were fertilized at the commercial facility on December 12, 2013, and reared as eggs in Heath stacks fed with chilled (3.9 ± 0.1°C) well water. Domesticated fish were transferred to the laboratory as mixed eyed-eggs/hatching alevin on March 23, 2014, at which point they were maintained in heath stacks until first feed as per T and W fish above. The D strain was generated in the mid 1980’s (approximately 11 generations under domestication pressure), and has been used in previous studies comparing physiological and genetic aspects of domestication including comparison to GH transgenic fish (e.g. [15, 41, 45]). At first feeding (April 14, 2014 for all groups, degree days = 835 for T and W fish, 690 for D fish), all fish from each genotype were pooled into stock populations. Prior to experimental setup, fish were transferred to multiple 15 L netted floating culture containers within 2000 L (T and W) or 200 L (D) tanks supplied with well water and fed sparingly with standard starter salmonid artificial food (Skretting Canada Ltd., Vancouver, BC).

The experiment was initiated on May 1, 2014 and lasted for eleven weeks. Initial weight and fork length was measured on 30 fish of each group that had been euthanized by an overdose of anaesthetic (200mg/L MS-222 [Syndel Laboratories, Nanaimo, BC] buffered with 400 mg/L sodium bicarbonate). For the experiment, fish groups were either reared together in naturalized stream tanks with or without predators, or reared separately in culture conditions as follows (see Fig 1). Culture conditions: 80 fish of each group were added to separate 15 L netted floating culture containers within aerated 200 L tanks (two containers of 80 fish per fish genotype, 1 container per 200 L tank). Naturalized stream tanks: Live fish (*n* = 80) from each of the three groups were moved to each of 8 different stream tanks (240 fish in total per stream tank). To distinguish between W and D fish, all W fish or all D fish within a tank, alternating between stream tanks, were adipose fin clipped. Clipping was performed by lightly anaesthetizing fry (50 mg/L MS-222 buffered with 100 mg/L sodium bicarbonate), removing the adipose fin, then allowing fish to recover before moving to the stream tanks. Stream tanks were long, shallow tanks (4.3 × 0.9 × 0.4 m) containing gravel bottoms and refuge areas (hollow rocks, logs, artificial plants), and covered with translucent plexiglass that allowed for natural lighting and minimized visual interactions between fish and care givers (as per [46]). Four of the stream tanks were designated as predation risk tanks. These tanks underwent staged predator attacks by a model great blue heron, once a day at random times per day, 5 days a week, for 4 weeks to allow for anti-predator behaviour in fish. The predator attacks were staged by lifting one section of the tank lid and plunging the head of a plastic model heron into the tank three times in rapid succession. Similar model avian attacks have been shown to elicit anti-predator behaviour in salmonids in both laboratory and natural conditions, e.g. [47–49]. Four weeks after the fish entered the stream tanks, three predatory fish (rainbow trout smolts, 75.3 ± 7.5 g, 20.2 ± 0.7 cm, that had been trained to feed on fry for three weeks prior to stream tank entry) were introduced to each of the four predator stream tanks.

Water for culture and stream tanks was directly supplied from nearby Cypress Creek. Water temperature over the experiment followed natural variation and ranged from 5.6–16.7°C (average 11.4 ± 0.3°C). Fish in stream tanks were fed natural food items (frozen bloodworms, mysis shrimp, and occasionally live juvenile crickets), once a day, 5 days a week, at random times in the day with food introduced randomly at one of three locations in each stream tank, to imitate unpredictable food availability potentially found in nature. In addition, natural prey items (e.g. stone fly, mayfly, and caddis fly larvae) were carried into the tanks through the unfiltered natural creek water supply. Fish in culture tanks were fed 4–6 times a day, 7 days a week, with standard salmonid artificial diets appropriate for the developmental stage (Skretting Canada Ltd., Vancouver, BC).

At eleven weeks from the start of the experiment, all fish were removed from the tanks, euthanized by an overdose of anaesthetic, weight and fork length determined, presence or absence of clip noted, and fins of all unclipped fish collected for genotyping to assess the presence or absence of the transgene. Crude amplifiable DNA from fin tissue was prepared by heating fin clips in 100 μL 0.01 N sodium hydroxide. The presence of the OnMTGH1 transgene was determined using a transgene-specific qPCR assay (see [50]). Fish from culture tanks were euthanized by an overdose of anaesthetic, enumerated, and weight and fork length of 30 fish per tank recorded.

### Data Analyses

Condition factor was calculated as: (weight in grams) × (fork length in cm)^-3^ × 100. Statistical analyses were performed using R V0.98.1103 ([51]). Differences among genotypes in initial log-weight, fork length, and condition factor were analyzed with the ANOVA function followed by paired t-tests. Generalized linear mixed models were used to test for differences in variables within culture tanks or semi-natural stream tanks using the lme4 package [52]. Within the culture tanks, genotype was a fixed factor with tank as a random variable [model: lmer (variable ~ Genotype + (1|Tank)]. Within semi-natural stream tanks, genotype, predation, and their interactions were included as fixed factors, and genotype was crossed with the random factor stream tank. Log-weight, fork length and condition factor were modelled using the normal distribution and identity link as [lmer (variable ~ Predator*Genotype + (1|Stream/Genotype)]. Survival was modelled using a binomial distribution and log-link function, adding the individual observations (i.e. tank) as a random factor to account for overdispersion: [glmer (survival ~ Predator*Genotype + (1|Stream/Genotype) + (1/obs)]. Significance of the model outputs were evaluated using the Anova() function in the car package with test type 3 [53]. *Post-hoc* comparisons between genotypes were evaluated using predicted values based on above models (predict() function in lme4 package) and paired t-tests. Presence of fin clip was originally included as a fixed factor in the stream tank models, but in all variables was found to be insignificant (weight: χ^2^ = 0.269, *P* = 0.604, length: χ^2^ = 0.464, *P* = 0.469, condition factor: χ^2^ = 0.026, *P* = 0.872, survival: χ^2^ = 0.188, *P* = 0.665), and was consequently excluded from the final models. Data are presented as mean ± standard error of the mean, and differences are considered significant if *P* < 0.05.

## Results

Raw data is given in S1 File. At the start of the experiment, T, D, and W fish did not differ significantly in weight (0.36 ± 0.01, 0.34 ± 0.02, 0.32 ± 0.02 g respectively, F_2,87_ = 1.812, *P* = 0.170, n = 30 fish) or length (3.56 ± 0.03, 3.23 ± 0.05, 3.30 ± 0.03 cm respectively, F_2,87_ = 2.947, *P* = 0.058), although W fish had a lower condition factor than D, but not T fish (0.86 ± 0.03, 0.98 ± 0.02, 0.94 ± 0.02 respectively, F_2,87_ = 6.609, *P* = 0.002).

**Fig 2:**
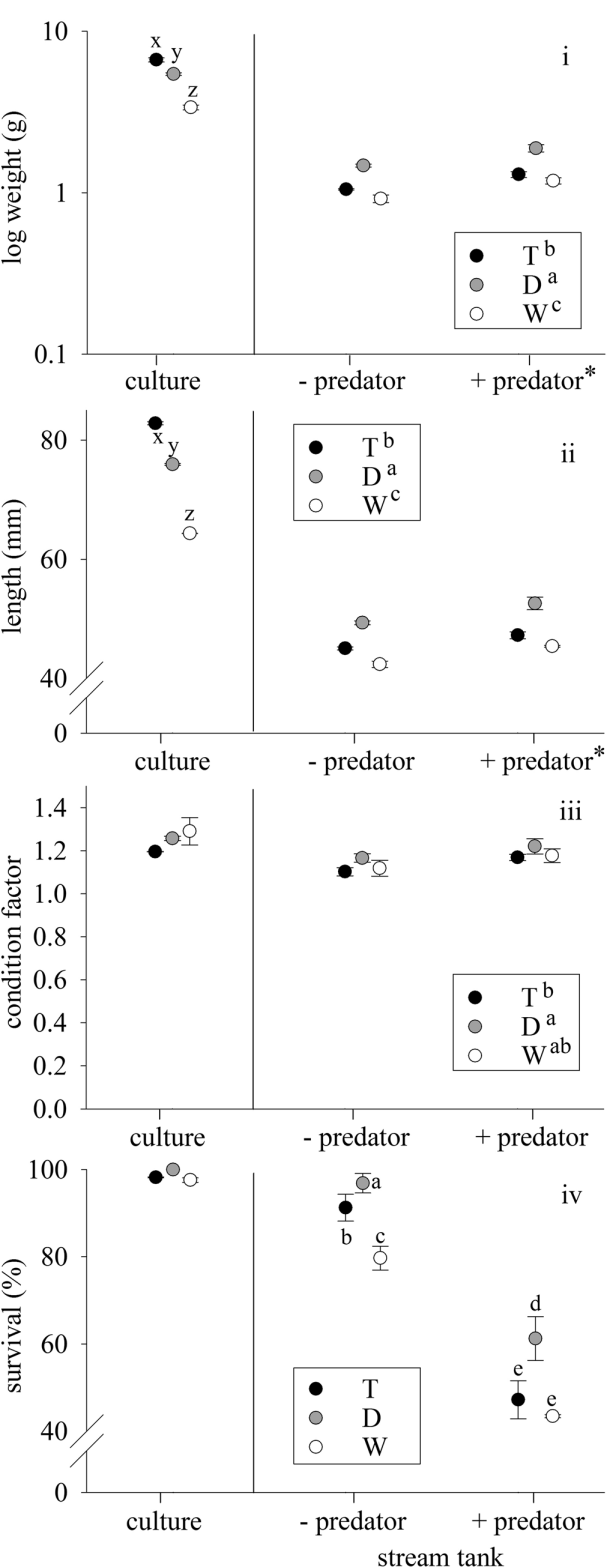
Genotype size, condition factor and survival. **i)** Log weight, **ii)** length, **iii)** condition factor, and **iv)** survival of growth hormone transgenic (T), domesticated (D), or wild-type (W) coho salmon fry after rearing in culture or semi-natural stream tanks without (-) or with (+) rainbow trout smolt predators. Data are given as means ± standard error of the mean. Letters indicates significant differences between genotypes within culture (x,y,z) or stream tanks (a,b,c,d,e). * indicates significant effect of predation.

After the growth period, both weight and length within culture conditions were in the order of T > D > W (*P* < 0.001 for both, χ^2^ = 102.7 and 239.4 respectively, Fig 2i and 2ii, Table 1). Condition factor in culture did not significantly differ between the three genotypes (χ^2^ = 2.82, *P* = 0.245, Fig 2iii, Table 1), nor did survival (χ^2^ = 0.103 *P* = 0.950, Fig 2iv, Table 1).

**Table 1:** Rank Order and Ratios of Differences Among Genotypes.

	Culture	Stream -Predator	Stream +Predator
**Weight**	T > D > W	D > T > W	D > T > W
	1.97: 1.61: 1	1.60: 1.14: 1	1.58: 1.09: 1
**Length**	T > D > W	D > T > W	D > T > W
	1.29: 1.18: 1	1.16: 1.06: 1	1.15: 1.04: 1
**CF**	W = D = T	D = W = T	D = W = T
	1: 0.97: 0.92	1.04: 1: 0.99	1.03: 1: 0.99
**Survival**	D = T = W	D > T > W	D > T = W
	1.03: 1.01: 1	1.22: 1.15: 1	1.41: 1.09: 1

Rank order and relative weight, length, condition factor (CF), and survival of domesticated (D), growth hormone transgenic (T), and wild-type (W) coho salmon, reared in culture conditions, or in naturalized stream tanks without (-) or with (+) predators. Ratios of variables are given in relation to wild-type fish, and > or < in rank order indicate significant differences among fish groups (*P* < 0.05).

In the stream tanks, there were no significant interactions between genotype and presence of predators in weight (χ^2^ = 0.531, *P* = 0.796), length (χ^2^ = 1.219, *P* = 0.544), or condition factor (χ^2^ = 0.463, *P* = 0.931). Genotypes differed in weight (χ^2^ = 124.0, *P* < 0.001, Fig 2i), length (χ^2^ = 129.0, *P* < 0.001, Fig 2ii), and condition factor (χ^2^ = 18.5, *P* < 0.001, Fig 2iii). For weight and length, genotype size ranked D > T > W, while D had greater condition factor than T fish overall (*P* = 0.042), but not when examined either in the absence or in the presence of predators (Fig 2iii and Table 1). Predation had a significant influence on weight (χ^2^ = 16.5, *P* < 0.001) and length (χ^2^ = 18.2, *P* < 0.001), but did not influence condition factor (χ^2^ = 1.63, *P* = 0.202). Fish were larger in the presence of predators than in their absence (Fig 2).

There was a significant interaction in survival between predation and genotype within the stream tanks (χ^2^ = 10.6, *P* = 0.005). Survival differed among all genotypes in the absence of predators (D > T > W, *P* range 0.006 to < 0.001), while in the presence of predators T did not differ from W in survival (*P* = 0.310), with D having higher survival than both T and W (*P* = 0.006 and 0.002 respectively, see Fig 2iv and Table 1).

## Discussion

The current study demonstrates that different types of anthropogenic-induced genetic change (i.e. selected versus engineered) used to achieve the same desired trait (i.e. accelerate growth) may differentially interact with environmental conditions to alter fitness components important in ecological success in fish. In the strains used, specific engineered genetic change (i.e. GH transgenesis) generated the largest phenotypic alteration (body size) and no effect on survival under conditions that maximize growth and survival (e.g. culture conditions with excess feed available and a lack of predators). In contrast, in more complex or challenging conditions, genetic change by domestication resulted in larger effects on body size and better survival than that observed in transgenic fish. As such, the genotypic change resulting in greatest change in growth in culture would not necessarily result in greatest growth and survival, and hence potential for ecological effects, in more natural conditions.

### Culture Environment

As expected, both transgenic and domesticated fry modified for greater growth grew to larger sizes than wild-type fish in culture. In these non-limiting food conditions, GH transgenesis resulted in greater size than artificial selection, as has been previously observed in these strains [41, 45]. The greater size in transgenic fish could be due to greater expression of GH and consequent effects on downstream pathways in transgenic versus domesticated individuals [41], resulting in greater maximum size at age. The domesticated strain used in the current study has a relatively recent origin (i.e. mid-1980s), and as such, the process of selection may not yet have maximized growth rate under culture conditions in this strain. As well, differences in early rearing (i.e. pre-hatch) between domesticated and transgenic fish may have contributed in part to differences in size as transgenic fish had greater number of pre-first feeding degree days than domesticated fish (835 versus 690).

### Semi-natural Environment Growth

Effects on body size and survival from transgenesis and domestication were significantly different in semi-natural stream tanks than culture conditions, where in these complex and limiting conditions, increased intrinsic growth through domestication resulted in greater maintenance of larger body size and higher survival, while size in the transgenic fish was more similar to that of wild type (see Fig 2, Table 1). These results are consistent with other studies where domesticated fish maintained high growth relative to wild-type fish in challenging environments to a greater extent than studies comparing transgenic and wild-type fish. For example, selection for high growth rate in Atlantic salmon (domestication) resulted in greater maintenance of growth (i.e. flatter reaction norms) than wild-type fish during challenging conditions (predation, decreased water level, high sediment load, [14, 54, 55]). As well, domesticated coho and Atlantic salmon maintained greater size than wild type whether reared in semi-natural or culture conditions [56, 57]. In contrast, growth rates of GH transgenic coho in previous studies were much closer to those of wild type when reared in semi-natural tanks than in culture conditions [16–18, 20], although transgenic coho salmon did maintain greater scope for growth than wild type in all but the most extreme conditions (e.g. [16]). The current results are in contrast to [21] who found GH transgenic rainbow trout fry had greater overwinter growth than domesticated x wild hybrids in a stream mesocosm. This may be due to wild-type genetic influence on growth in the domesticated-hybrid genome (e.g. [13, 15]), or due to differences in seasonal growth rates between transgenic and domesticated × wild salmonids (e.g. transgenic coho salmon continue growing over winter in culture, while domesticated and domesticated x wild hybrids may not [58, 59]).

The specific environmental factors that influence differential shifts in growth among the groups of fish in culture versus semi-natural environments are not known, but may include differences in feed type and abundance, habitat complexity, and fish density. Strain effects were likely not a strong influence in the current study, as size ratio of domesticated versus wild type was similar across environments, while transgenic versus wild type was not (see Table 1), even though transgenic fish, and not domesticated fish, were of the same strain as, and shared maternal parents with, wild-type fish. As the parental fish used for both T and W fish (Chehalis River Hatchery strain) likely had mixed hatchery and wild recent ancestory, inadvertant selection for increased fitness in hatchery versus natural conditions as juveniles may have taken place, and has been reported to decrease survival to predation in steelhead salmon fry [60]. However, the hatchery strain used in the current study displays typical wild juvenile behaviour in culture (balancing foraging behaviour with predator avoidance, see Supplemental Video in [4]), indicating wild-type behavioural phenotype is maintained to some extent in this strain. Differences in genotype size rank order between culture and stream-tank growth rates may have also been due to genotypes responding differently to being reared seperately (culture tanks) or together (stream tanks), although [57] found there was no difference in relative growth rates of D and W Atlantic salmon when reared together or seperately in culture conditions. In hatchery conditions, co-rearing can differentially influence growth of W and/or T fish relative to rearing alone when food is limiting, indicating strong competitive effects can act to influence phenotype under some simple conditions [61].

### Semi-natural Environment Survival

Domesticated and GH transgenic fry had greater survival than wild-type fry in stream tanks without predation, with domesticated fish having highest survival of any genotype. This may be due to greater foraging and aggressive behaviour observed in growth-enhanced fish [9, 12, 13, 23, 26, 31–33] resulting in a greater proportion of the growth-enhanced fish obtaining adequate food for survival in these limiting conditions, particularly for domesticated fish. This concurs with other studies that found that domesticated or transgenic fish can negatively impact fitness of wild or laboratory populations [8, 10, 61, 62]. As well, cannibalism of wild-type fish by growth-enhanced fish may have occurred once size disparity between the groups was established, as has been observed in similar experiments [13, 61]. In the presence of predators, domesticated fish had highest survival, while transgenic fish had survival similar to wild-type fish (see Fig 2, Table 1). The genotype rank order of survival with predation is contrary to expected, as both domesticated and transgenic fish are reported in some circumstances to have greater mortality from predation than wild-type fish [8, 11, 13, 20, 63, 64]. However, transgenic fish may have higher predator mortality only when predators are present at first feeding [17, 21], or only with specific predator types [16]. Domesticated and, to a lesser extent, transgenic fish may have established themselves in the semi-natural conditions to a degree that counteracted the low predator avoidance behaviours observed in these strains (e.g. high position in water column, fast recovery to foraging behaviour after predator strike, [15, 24]). As well, wild-type coho salmon decrease predator avoidance behaviour in competitive and limiting environments [65], and small coho fry are reported to resume foraging sooner than larger fry after a predator attack [49], which may have increased susceptibility of the smaller wild-type fry to predation under competition. The high survival rate of domesticated fish in the presence of predators may have also been due to the size advantage of these fish over transgenic and wild-type fish resulting in decreased relative size-related predation, as has been observed in domesticated Atlantic salmon parr [14]. The larger size of fish under predation pressure may indicate smaller fish were preferentially preyed upon. However, larger size observed under predation conditions may also be due to lower numbers of fish resulting in greater food availability for remaining fish, or a combination of these two processes. These data indicate that domesticated fish may have increased potential for growth and survival relative to transgenic and wild-type fish, and have greater potential for ecological consequences, at least in environments similar to the semi-natural conditions presented here.

### Potential Genetic Basis for Genotype × Environment Interactions

The shift in body size and survival order and ratios of transgenic and domesticated fish in different environments (see Table 1) indicate these two genotypes responded to environmental conditions in different ways (i.e. genotype × environment interactions are present). This may be due to differences in the genetic basis for accelerated growth and behaviour in the two groups of fish. Previous studies have identified the GH/IGF-I axis as a mechanism for enhanced growth in domesticated and GH transgenic salmon [41, 42, 56, 66–68], and a potential mechanism for altered foraging, aggressive, and/or anti-predator behaviours [28, 29, 31, 69, 70]. In wild-type fish, GH expression is primarily in the pituitary gland and, along with downstream factors such as IGF-I, is highly regulated in response to physiological and environmental factors. In the transgenic fish strain utilized, GH is expressed in many tissues in a relatively unregulated way, although expression of some other growth-related genes (including IGF-I) were similar to that of wild-type fish when fed a wild-type ration ([45, 68]), suggesting wild-type physiological and/or environmental regulation of non-GH growth-related factors may exist in these fish. In contrast, increased GH expression in the current strain of domesticated coho salmon has only been noted in a non-pituitary tissue (muscle) thus far [41], and unlike wild-type fish, domesticated fry did not decrease plasma IGF-I in response to semi-natural stream tank rearing and limited-food conditions [56]. This leads to the hypothesis that in transgenic fish, the single genetic change of increased GH results in maximum growth in unlimiting conditions, but wild-type environmental and physiological regulation of other growth factors can result in growth similar to wild type in challenging conditions. In contrast, domestication may allow for altered environmental and physiological control of multiple growth factor pathways as well as concurrant counterselection against secondary (pleitropic) effects of accelerated growth, that could allow for maintainence of growth rate and survival in varied environmental conditions. In addition, domestication would select for fast growth in culture conditions which, although different from nature, are nevertheless still dynamic and would select for maintaining capacity and plasticity to grow under variable environmental conditions. Due to recent synthesis and continued back-crossing to wild-reared populations, transgenic fish of the strain examined here would not have had an opportunity for counterselection against the complex pleiotropic effects of increased GH, or for selection for fast growth in varied environments, which may result in them being less well adapted for fast growth in complex environments. Direct comparisons of multiple growth pathways in varying environments are needed to identify the mechanisms influencing differential growth potential of domesticated and GH transgenic fish.

## Conclusions

All three genotypes showed altered relative body size and growth in different environmental conditions. As size of domesticated fish was less affected by environment than transgenic fish, this could suggest domesticated fish were less capable of differentially responding in growth to different environmental conditions than transgenic fish. However, the higher survival of domesticated fish, with or without predators, indicates that the greater maintenance of high growth in naturalized conditions was a beneficial phenotype in these environments, rather than a detrimental lack of plasticity. While transgenic fish had a high growth rate in culture that was unachievable by domesticated fish, the lower survival and growth of transgenic fish in complex conditions indicate that the accelerated growth potential of these fish did not constitute an advantage in complex conditions relative to domesticated fish. It should be noted that significant differences in growth and survival between strains has been noted in domesticated (e.g. [19]) and transgenic (e.g. [44]) fish, and whether similar results would be obtained in other strains of domesticated and transgenic salmonids should be addressed. As well, the consequences of multi-generational introgression of the two growth-enhanced genotypes into wild populations would follow very different functional pathways, as phenotypic expression of domesticated traits are diluted with repeated crossing with wild type (e.g. [15]), while inheritability of transgenic traits into a wild population follow an all-or-nothing process (i.e. offspring either inherit the transgene or they do not).

The genetic basis for phenotypic plasticity in growth likely plays an important role in influencing fitness of organisms in nature, and anthropogenic-induced alterations in this capacity could affect the ability of an introduced strain to persist in and interact with ecosystem components. As such, the present data have implications for the potential of GH transgenic and domesticated salmon to survive in natural conditions and cause ecological consequences, and demonstrates that under laboratory conditions at least, assessed ecological consequences of one type of engineered growth-enhanced fish may not be extrapolated to other growth-enhanced fish, or between rearing environments, with reasonable certainty.

## Supporting Information

S1 File Raw size, growth and survival data of genotypes. Individual fish weight and length, and remaining fish numbers (for survival) used to calculate means ± standard error of the means of genotypes within environmental conditions.
(XLS)

## References

[1] Devlin RH, Biagi CA, Yesaki TY, Smailus DE, Byatt JC. Growth of domesticated transgenic fish. Nature. 2001;409: 781–2. 11236982 10.1038/35057314

[2] Gjedrem T, Robinson N, Rye M. The importance of selective breeding in aquaculture to meet future demands for animal protein: A review. Aquaculture. 2012;350–353: 117–29. 10.1016/j.aquaculture.2012.04.008

[3] Hershberger WK, Myers JM, Iwamoto RN, McAuley WC, Saxton AM. Genetic changes in the growth of coho salmon (*Oncorhynchus kisutch*) in marine net-pens, produced by ten years of selection. Aquaculture. 1990;85: 187–97.

[4] Devlin RH, Sundström LF, Leggatt RA. Assessing ecological and evolutionary consequences of growth-accelerated genetically engineered fishes. Bioscience. 2015;65: 685–700.

[5] Morris MRJ, Fraser DJ, Heggelin AJ, Whoriskey FG, Carr JW, O'Neil SF, et al Prevalence and recurrence of escaped farmed Atlantic salmon (*Salmo salar*) in eastern North American rivers. Can J Fish Aquat Sci. 2008;65: 2807–26. 10.1139/f08-181

[6] Bourret V, O'Reilly PT, Carr JW, Berg PR, Bernatchez L. Temporal change in genetic integrity suggests loss of local adaptation in a wild Atlantic salmon (*Salmo salar*) population following introgression by farmed escapees. Heredity. 2011;106: 500–10. 10.1038/hdy.2010.165 21224876 PMC3131974

[7] Berg S, Jorgensen J. Stocking experiments with 0+ and 1+ trout parr, *Salmo trutta* L, of wild and hatchery origin. 1. Post-stocking mortality and smolt yield. J Fish Biol. 1991;39: 151–69. 10.1111/j.1095-8649.1991.tb04353.x

[8] Fleming IA, Hindar K, Mjølnerød IB, Jonsson B, Balstad T, Lamberg A. Lifetime success and interactions of farm salmon invading a native population. Proc R Soc Lond Biol. 2000;267: 1517–23. 10.1098/rspb.2000.1173 PMC1690700 11007327

[9] Einum S, Fleming IA. Genetic divergence and interactions in the wild among native, farmed and hybrid Atlantic salmon. J Fish Biol. 1997;50: 634–51.

[10] McGinnity P, Prodöhl P, Ferguson A, Hynes R, Maoiléidigh NÓ, Baker N, et al Fitness reduction and potential extinction of wild populations of Atlantic salmon, *Salmo salar*, as a result of interactions with escaped farm salmon. Proc R Soc Lond Biol. 2003;270: 2443–50. 10.1098/rspb.2003.2520 PMC1691531 14667333

[11] Vandersteen WE, Biro P, Harris L, Devlin R. Introgression of domesticated alleles into a wild trout genotype and the impact on seasonal survival in natural lakes. Evol Appl. 2012;5: 76–88. 10.1111/j.1752-4571.2011.00210.x 25568031 PMC3353333

[12] Blann CA, Healey MC. Effects of species, culture history, size and residency on relative competitive ability of salmonids. J Fish Biol. 2006;69: 535–52.

[13] Tymchuk WE, Sundström LF, Devlin RH. Growth and survival trade-offs and outbreeding depression in rainbow trout (*Oncorhynchus mykiss*). Evolution. 2007;61: 1225–37. 17492973 10.1111/j.1558-5646.2007.00102.x

[14] Debes PV, Hutchings JA. Effects of domestication on parr maturity, growth, and vulnerability to predation in Atlantic salmon. Can J Fish Aquat Sci. 2014;71: 1371–84. 10.1139/cjfas-2013-0618

[15] Tymchuk WE, Biagi C, Withler R, Devlin RH. Growth and behavioral consequences of introgression of a domesticated aquaculture genotype into a native strain of coho salmon. Trans Am Fish Soc. 2006;135: 442–55.

[16] Sundström LF, Devlin RH. Increased intrinsic growth rate is advantageous even under ecologically stressful conditions in coho salmon (*Oncorhynchus kisutch*). Evol Ecol. 2011;25: 447–60. 10.1007/s10682-010-9406-1

[17] Sundström LF, Lõhmus M, Devlin RH. Selection on increased intrinsic growth rates in coho salmon, *Oncorhynchus kisutch*. Evolution. 2005;59: 1560–9. 16153041

[18] Sundström LF, Tymchuk WE, Lõhmus M, Devlin RH. Sustained predation effects of hatchery-reared transgenic coho salmon *Oncorhynchus kisutch* in semi-natural environments. J Appl Ecol. 2009;46: 762–9. 10.1111/j.1365-2664.2009.01668.x

[19] Skaala Ø, Glover KA, Barlaup BT, Svåsand T, Besnier F, Hansen MM, et al Performance of farmed, hybrid, and wild Atlantic salmon (*Salmo salar*) families in a natural river environment. Can J Fish Aquat Sci. 2012;69: 1994–2006.

[20] Sundström LF, Lõhmus M, Johnsson JI, Devlin RH. Growth hormone transgenic salmon pay for growth potential with increased predation mortality. Proc R Soc Lond Biol. 2004;271: S350–S2. 10.1098/rsbl.2004.0189 PMC1810071 15504015

[21] Crossin GT, Sundström LF, Vandersteen WE, Devlin RH. Early life-history consequences of growth-hormone transgenesis in rainbow trout reared in stream ecosystem mesocosms. PLoS ONE. 2015;10: e0120173 10.1371/journal.pone.0120173 25807001 PMC4373795

[22] Tymchuk WE, Devlin RH, Withler RE. The role of genotype and environment in phenotypic differentiation amoung wild and cultured salmonids In: DFO, editor. A scientific review of the potential environmental effects of aquaculture in aquatic ecosystems Vol IV Canadian Technical Report of Fisheries and Aquatic Sciences 2450. Ottawa, ON: Fisheries and Oceans Canada Science Sector; 2006 p. 1–58.

[23] Sundström LF, Lõhmus M, Devlin RH, Johnsson JI, Biagi CA, Bohlin T. Feeding on profitable and unprofitable prey: Comparing behaviour of growth-enhanced transgenic and normal coho salmon (*Oncorhynchus kisutch*). Ethology. 2004;110: 381–96.

[24] Sundström LF, Devlin RH, Johnsson JI, Biagi CA. Vertical position reflects increased feeding motivation in growth hormone transgenic coho salmon (*Oncorhynchus kisutch*). Ethology. 2003;109: 701–12.

[25] Houde ALS, Fraser DJ, Hutchings JA. Reduced anti-predator responses in multi-generational hybrids of farmed and wild Atlantic salmon (*Salmo salar* L.). Conserv Genet. 2010;11: 785–94.

[26] Sundström LF, Lõhmus M, Johnsson JI, Devlin RH. Dispersal potential is affected by growth-hormone transgenesis in coho salmon (*Oncorhynchus kisutch*). Ethology. 2007;113: 403–10.

[27] Drew RE, Settles ML, Churchill EJ, Williams SM, Balli S, Robison BD. Brain transcriptome variation among behaviorally distinct strains of zebrafish (*Danio rerio*). BMC Genomics. 2012;13: 323 10.1186/1471-2164-13-323 22817472 PMC3434030

[28] Johnsson JI, Petersson E, Jönsson E, Björnsson BT, Järvi T. Domestication and growth hormone alter antipredator behaviour and growth patterns in juvenile brown trout, *Salmo trutta*. Can J Fish Aquat Sci. 1996;53: 1546–54.

[29] Jönsson E, Johnsson JI, Björnsson BT. Growth hormone increases aggressive behavior in juvenile rainbow trout. Horm Behav. 1998;33: 9–15. 10.1006/hbeh.1997.1426 9571008

[30] Ruzzante DE. Domestication effects on aggressive and schooling behavior in fish. Aquaculture. 1994;120: 1–24. 10.1016/0044-8486(94)90217-8

[31] Devlin RH, Johnsson JI, Smailus DE, Biagi CA, Jönsson E, Björnsson BT. Increased ability to compete for food by growth hormone-transgenic coho salmon *Oncorhynchus kisutch* (Walbaum). Aquacult Res. 1999;30: 479–82.

[32] Houde ALS, Fraser DJ, Hutchings JA. Fitness-related consequences of competitive interactions between farmed and wild Atlantic salmon at different proportional representations of wild–farmed hybrids. ICES J Mar Sci. 2010;67: 657–67.

[33] Metcalfe NB, Valdimarsson SK, Morgan IJ. The relative roles of domestication, rearing environment, prior residence and body size in deciding territorial contests between hatchery and wild juvenile salmon. J Appl Ecol. 2003;40: 535–44.

[34] Moreau DTR, Fleming IA, Fletcher GL, Brown JA. Growth hormone transgenesis does not influence territorial dominance or growth and survival of first-feeding Atlantic salmon *Salmo salar* in food-limited stream microcosms. J Fish Biol. 2011;78: 726–40. 10.1111/j.1095-8649.2010.02888.x 21366569

[35] Moreau DTR, Gamperl AK, Fletcher GL, Fleming IA. Delayed phenotypic expression of growth hormone transgenesis during early ontogeny in Atlantic salmon (*Salmo salar*)? PLoS ONE. 2014;9: e95853 10.1371/journal.pone.0095853 24763675 PMC3998944

[36] Solberg MF, Zhang ZW, Glover KA. Are farmed salmon more prone to risk than wild salmon? Susceptibility of juvenile farm, hybrid and wild Atlantic salmon *Salmo salar* L. to an artificial predator. Appl Anim Behav Sci. 2015;162: 67–80. 10.1016/j.applanim.2014.11.012

[37] Sundström LF, Lõhmus M, Devlin RH. Migration and growth potential of coho salmon smolts: implications for ecological impacts from growth-enhanced fish. Ecol Appl. 2010;20: 1372–83. 10.1890/09-0631.1 20666255

[38] Harwood AJ, Armstrong JD, Metcalfe NB, Griffiths SW. Does dominance status correlate with growth in wild stream-dwelling Atlantic salmon (*Salmo salar*)? Behav Ecol. 2003;14: 902–8. 10.1093/beheco/arg080

[39] Sloman KA, Armstrong JD. Physiological effects of dominance hierarchies: laboratory artefacts or natural phenomena? J Fish Biol. 2002;61: 1–23. 10.1006/jfbi.2002.2038

[40] Adriaenssens B, Johnsson JI. Shy trout grow faster: exploring links between personality and fitness-related traits in the wild. Behav Ecol. 2011;22: 135–43. 10.1093/beheco/arq185

[41] Devlin RH, Sakhrani D, Tymchuk WE, Rise ML, Goh B. Domestication and growth hormone transgenesis cause similar changes in gene expression in coho salmon (*Oncorhynchus kisutch*). PNAS. 2009;106: 3047–52. 10.1073/pnas.0809798106 19223591 PMC2651260

[42] Devlin RH, Sakhrani D, White S, Overturf K. Effects of domestication and growth hormone transgenesis on mRNA profiles in rainbow trout (*Oncorhynchus mykiss*). J Anim Sci. 2013;91: 5247–58. 10.2527/jas.2013-6612 24045478

[43] Devlin RH, Yesaki TY, Biagi CA, Donaldson EM, Swanson P, Chan W-K. Extraordinary salmon growth. Nature. 1994;371: 209–10.

[44] Devlin RH, Biagi CA, Yesaki TY. Growth, viability and genetic characteristics of GH transgenic coho salmon strains. Aquaculture. 2004;236: 607–32. 10.1016/j.aquaculture.2004.02.026

[45] Overturf K, Sakhrani D, Devlin RH. Expression profile for metabolic and growth-related genes in domesticated and transgenic coho salmon (*Oncorhynchus kisutch*) modified for increased growth hormone production. Aquaculture. 2010;307: 111–22. 10.1016/j.aquaculture.2010.06.010

[46] Sundström LF, Lõhmus M, Tymchuk WE, Devlin RH. Gene-environment interactions influence ecological consequences of transgenic animals. PNAS. 2007;104: 3889–94. 10.1073/pnas.0608767104 17360448 PMC1820679

[47] Johnsson JI. Group size influences foraging effort independent of predation risk: an experimental study on rainbow trout. J Fish Biol. 2003;63: 863–70. 10.1046/j.1095-8649.2003.00187.x

[48] Dionne M, Dodson JJ. Impact of exposure to a simulated predator (*Mergus merganser*) on the activity of juvenile Atlantic salmon (*Salmo salar*) in a natural environment. Can J Zool. 2002;80: 2006–13. 10.1139/z02-176

[49] Reinhardt UG, Healey MC. Season- and size-dependent risk taking in juvenile coho salmon: experimental evaluation of asset protection. Anim Behav. 1999;57: 923–33. 10.1006/anbe.1998.1051 10202100

[50] Devlin RH, Sakhrani D, Biagi CA, Eom K-W. Occurrence of incomplete paternal-chromosome retention in GH-transgenic coho salmon being assessed for reproductive containment by pressure-shock-induced triploidy. Aquaculture. 2010;304: 66–78.

[51] R Core Team. R: A language and environment for statistical computing Vienna, Austria R Foundation for Statistical Computing, 2015 URL http://www.R-project.org/.

[52] Bates D, Maechler M, Bolker B, Walker S. Lme4: Linear mixed-effect models using Eigen and S4. R package version 1.17. 2014. http://CRAN.R-project.org/package=lme4.

[53] Fox J, Weisberg S. An R Companion to Applied Regression, Second Edition Thousand Oaks, CA: Sage Publications, Inc.; 2011. 472 p.

[54] Solberg MF, Skaala Ø, Nilsen F, Glover KA. Does domestication cause changes in growth reaction norms? A study of farmed, wild and hybrid Atlantic salmon families exposed to environmental stress. PLoS ONE. 2013;8: e54469 10.1371/journal.pone.0054469 23382901 PMC3561353

[55] Debes PV, Fraser DJ, Yates M, Hutchings JA. The between-population genetic architecture of growth, maturation, and plasticity in Atlantic salmon. Genetics. 2014;196: 1277–91. 10.1534/genetics.114.161729 24473933 PMC3982675

[56] Tymchuk WE, Beckman B, Devlin RH. Altered expression of growth hormone/insulin-like growth factor I axis hormones in domesticated fish. Endocrinology. 2009;150: 1809–16. 10.1210/en.2008-0797 19022885

[57] Solberg MF, Zhang A, Nilsen F, Glover KA. Growth reaction norms of domesticated, wild and hybrid Atlantic salmon families in response to differeing social and physical environments. BMC Genomics. 2013;13: 234. 10.1186/1471-2148-13-234 PMC4231500 24165438

[58] Oakes JD, Higgs DA, Eales JG, Devlin RH. Influence of ration level on the growth performance and body composition of non-transgenic and growth-hormone-transgenic coho salmon (*Oncorhynchus kisutch*). Aquaculture. 2007;265: 309–24. 10.1016/j.aquaculture.2007.01.015

[59] Johnsson JI, Clarke WC, Withler RE. Hybridization with domesticated rainbow trout (*Oncorhynchus mykiss*) reduces seasonal variation in growth of steelhead trout (*O*. *mykiss*). Can J Fish Aquat Sci. 1993;50: 480–7.

[60] Berejikian BA. The effects of hatchery and wild ancestry and experience on the relative ability of steelhead trout fry (*Oncorhynchus mykiss*) to avoid a benthic predator. Can J Fish Aquat Sci. 1995;52: 2476–82. 10.1139/f95-838

[61] Devlin RH, D'Andrade M, Uh M, Biagi CA. Population effects of growth hormone transgenic coho salmon depend on food availability and genotype by environment interactions. PNAS. 2004;101: 9303–8. 15192145 10.1073/pnas.0400023101 PMC438972

[62] Sundström LF, Vandersteen WE, Lõhmus M, Devlin RH. Growth-enhanced coho salmon invading other salmon species populations: effects on early survival and growth. J Appl Ecol. 2014;51: 82–9. 10.1111/1365-2664.12185

[63] Dieperink C, Pedersen S, Pedersen MI. Estuarine predation on radiotagged wild and domesticated sea trout (*Salmo trutta* L.) smolts. Ecol Freshw Fish. 2001;10: 177–83.

[64] Biro PA, Abrahams MV, Post JR, Parkinson EA. Predators select against high growth rates and risk-taking behaviour in domestic trout populations. Proc R Soc Lond Biol. 2004;271: 2233–7. 10.1098/rspb.2004.2861 PMC1691858 15539348

[65] Dill LM, Fraser AHG. Risk of predation and the feeding behavior of juvenile coho salmon (*Oncorhynchus kisutch*). Behav Ecol Sociobiol. 1984;16: 65–71. 10.1007/bf00293105

[66] Fleming IA, Agustsson T, Finstad B, Johnsson JI, Björnsson BT. Effects of domestication on growth physiology and endocrinology of Atlantic salmon (*Salmo salar*). Can J Fish Aquat Sci. 2002;59: 1323–30. 10.1139/F02-082

[67] Wringe BF, Devlin RH, Ferguson MM, Moghadam HK, Sakhrani D. Growth-related quantitative trait loci in domestic and wild rainbow trout (*Oncorhynchus mykiss*). BMC Genomics. 2010;11: 63 10.1186/1471-2156-11-63 20609225 PMC2914766

[68] Raven PA, Uh M, Sakhrani D, Beckman BR, Cooper K, Pinter J, et al Endocrine effects of growth hormone overexpression in transgenic coho salmon. Gen Comp Endocrin. 2008;159: 26–37. 10.1016/j.ygcen.2008.07.011 18713628

[69] Abrahams MV, Sutterlin A. The foraging and antipredator behaviour of growth-enhanced transgenic Atlantic salmon. Anim Behav. 1999;58: 933–42. 10564595 10.1006/anbe.1999.1229

[70] Neregård L, Sundt-Hansen L, Björnsson BT, Johnsson JI. Growth hormone affects behaviour of wild brown trout *Salmo trutta* in territorial owner-intruder conflicts. J Fish Biol. 2008;73: 2341–51. 10.1111/j.1095-8649.2008.02082.x

